# Risk Influence of Some Environmental and Behavioral Factors on Air Contamination in the Operating Room: An Experimental Study

**DOI:** 10.3390/ijerph20166592

**Published:** 2023-08-17

**Authors:** Prospero Albertini, Pierangela Mainardi, Maria Bagattini, Annalisa Lombardi, Patrizia Riccio, Maria Ragosta, Francesca Pennino, Dario Bruzzese, Maria Triassi

**Affiliations:** 1 Department of Public Health, University “Federico II”, Via Sergio Pansini N° 5, 80131 Naples, Italy; prosperoal@libero.it (P.A.); pierangela.mainardi@gmail.com (P.M.); maria.bagattini@unina.it (M.B.); annalisa.lombardi@unina.it (A.L.); dario.bruzzese@unina.it (D.B.); maria.triassi@unina.it (M.T.); 2 Department of Molecular Medicine and Medical Biotechnology, University “Federico II”, Via Sergio Pansini N° 5, 80131 Naples, Italy; pariccio@unina.it; 3 School of Engineering, University of Basilicata, V.le dell’Ateneo Lucano N° 10, 85100 Potenza, Italy; maria.ragosta@unibas.it

**Keywords:** pollution in the operating theatres, measurement protocol, airborne microorganisms, dust concentration, surgical masks, doors opening in operating theatres

## Abstract

Air contamination in operating rooms (ORs) depends on the conditions of the room and on activities therein performed. Methodologies of air quality assessment in ORs are often inadequately described in the scientific literature, and the time required for a change in status in air quality is never taken into account. The purpose of this study was to determine the influence of the state and the presence of human operators on air quality by implementing a precise measurement protocol that also took into account the time required for changes in the room to affect air pollution. As the main indicators of air pollution, bacterial load and concentration of airborne dust were measured. The results showed that: the use of surgical masks by operators in the OR did not significantly affect bacterial load within a distance of 2 m; keeping OR doors open did not induce a significant increase in bacterial load and of 5 μm particles while 10 μm particles concentration was positively affected; and air pollution measured with open doors was not significantly different from that due to the presence of two staff members, whether or not they were wearing masks. The results clarified the role of some factors on air pollution in ORs.

## 1. Introduction

Air quality in operating rooms (ORs) is a factor to be monitored to prevent infections in hospital patients [1]. Microbiological contamination is principally due to the presence of staff, patients, all the different surgical objects in the ORs and the different activities performed in the place [2]. 

The bacteria most commonly detected in surgical site infections (SSIs) are coagulase-negative *Staphylococci* and *Mycobacterium chimaera*, and the bacterium that causes most SSIs in ORs is *Staphylococcus aureus* [3].

Conditions that can result in a greater control of microbiological contamination in a room are high ventilation rates and mixing ventilation strategies. Other factors that may determine such control are air changes per hour and the position of the vents [4].

Several studies [1,5,6,7,8,9,10,11,12,13,14] evaluated air quality in the Ors in various conditions to determine how such conditions could influence air quality.

These studies had some limitations; for example, they are missing some experimental conditions, such as an initial evaluation of the ventilation system and its efficiency, location of detection tools during air sampling, description of sampling time, presence and disposition of the ORs staff, the use or not of surgical masks, and the status of doors at time of sampling. In addition, these studies did not take into account the introduction or the elimination of a pollution source and the modification of its emissions, necessary conditions to allow the stabilization of the contaminants concentration in the air of the OR.

A systematic procedure was developed in a work by Tan et al. to establish the relationship between particulate matter (PM) and microbial counts in OR in Malaysia. The study showed no positive correlation between microbial count and PM 0.5 and a clear correlation of 7% with PM5 and 15% with PM10, suggesting constant monitoring of these two parameters prior to each surgical procedure [15].

In Italy, the ISPESL (National Institute for Occupational Safety and Prevention) Guidelines [16] (that provide information on objectives, sampling methods, sampling frequency, and interpretation of results) and the International Standard Organization (ISO) are available to control contamination in ORs [17].

The aim of this study was to assess how specific conditions can influence air quality in the OR. Different conditions, such as the presence or not of the staff, the use or not of surgical masks, and the status of the OR doors (opened or closed), were tested. Moreover, a strict measurement protocol was applied that, after the control of the air exchange system conditions, would also evaluate the time required to achieve a stationary condition for the air pollutants when experimental condition changed.

In relation to the effects associated to the variability of these conditions during the normal use of the ORs, these variables, from case to case, associated with different factors related with the surgery itself, number of staff members in the room, use of the OR equipment, and so on were taken into account. In addition, when changing these conditions during each single surgery, the effects of their variability can overlap, determining a real impossibility to discern between the changes related to the studied variables and those associated with others. For this reason, this study was carried out in ORs in disuse, modifying one condition at a time and waiting for the environment to reach a stationary state.

## 2. Materials and Methods

### 2.1. Setting: ORs

The study was conducted at the “University Hospital Federico II” in Naples, Italy. All data were collected in 12 ORs, each of which had a surface of approximately 30 m^2^ and a volume of 100 m^3^. In order to achieve uniformity in this study, all tests were performed in General Surgery Ors.

All Ors had ISO class 7 indoor environment. These Ors maintained compliance with hygienic-environmental requirements and physical and chemical parameters, such as ventilation, differential pressure, microclimatic parameters, concentration of airborne anesthetic agents, etc. These conditions determine whether activities in the OR are carried out safely [16].

In addition, all ORs had a ventilation system with a turbulent flow blown out from a diffuser positioned in the center of the ceiling. Sucked air was collected from nozzles positioned at the bottom of the walls so as to create a continuous centrifugal air flow from the center of the room towards the walls. The presence of these elements suggested the existence of a ventilation system with a turbulent flow. Another factor that suggested this condition was that these rooms were all General Surgery ORs (the ISPESL Guidelines recommend this type of flow for these ORs).

The air flows were set in order to create a slight positive pressure related to the surrounding environment. The system allowed a minimum of 15 air exchanges per hour and was equipped with HEPA filters able to remove 99.99% of the particles floating in the air with a dimension ≥0.3 µm in order to guarantee an air quality in accordance with Class 7 of the ISO 14644-1:2015 [18]. Temperature and humidity of the air were kept between 20–24 °C and 40–60%, respectively.

### 2.2. Air Sampling and Analysis

The assessment of air pollution was achieved by determining both bacterial load and numbers of airborne particles in the air. For each OR, air quality was evaluated with microbiological sampling of the air and airborne particles counting with an aerodynamic diameter (diameter correspondent to that of a sphere having a specific mass equal to 1 and with the same aerodynamic behavior) of ≥5 µm (5 µm) and ≥10 µm (10 µm).

Air sampling and subsequent analyses were performed by our laboratory staff. Our laboratory is accredited according to ISO/IEC 17025:2017 [19] and periodically performs proficiency tests. The equipment used for the study was periodically calibrated.

Airborne microorganisms were assessed according to ISO 14698-1:2003 [17] and EN 13098:2019 [20]. The active air sampling was performed using the Surface Air System Sampler SAS Super ISO 180 (VWR PBI International, Milan, Italy), with 55-mm diameter RODAC (Replicate Organism Direct Agar Contact) containing PCA (Plate Count Agar) (Oxoid Italia SpA, Milan, Italy), with flow rate of 180 L/min and suction volume set to 1000 L. The collection time of the air samples was 5 min. Air sampling was performed at 1 m from the floor. 

The Total Viable Count (TVC) in the air was detected in accordance with ISO 4833-2:2013 [21]. The PCA plates were incubated at a temperature of 30 ± 1 °C for 72 h. After incubation, the number of CFUs (colony-forming units) was adjusted using the conversion table provided by the manufacturer and was expressed as CFU/m^3^. 

The number of airborne particles was carried out following the European Regulations ISO 14644-1:2015 [18] and using the discrete airborne particles counter Lighthouse SOLAIR 3100 GenE (AM Instments Srl, Limbiate, Italy) compliant with the standards of ISO 21501-4:2018 [22]. The result was expressed as number of particles (N)/m^3^.

Each sampling had a duration of 5 min for a total air aspiration of 140 L so as to obtain a statistically significant sample of at least 20 particles/m^3^ (10 µm particles) for Class 7 of ISO 14644-1:2015 [18] for OR.

### 2.3. Operative Protocol

Before carrying out the study, the ventilation system efficiency in the OR was evaluated, measuring the speed of the air blown out by the diffusers and calculating the number of air exchanges.

To evaluate this number, an anemometer connected to a microclimatic station Babuc (LSI Lastem, Milan, Italy) was used, in accordance to EN ISO 7726:2002 [23].

All ORs that had a microbial load in the blown air >2 CFU/m^3^ and a concentration of 5 µm and 10 µm airborne particles more than 2930 N/m^3^ and 692 N/m^3^ respectively, were excluded, in accordance with Class 7 of ISO 14644-1:2015 [18] (higher values are indicative of filters or air conditioning system malfunction). Based on the obtained results, 2 ORs from the study were excluded; therefore, our study was carried out in only 10 ORs.

All measurements in ORs were conducted with the presence or not of staff members, with or without the use of masks, with opened or closed doors, and positioning the detection equipment in the most important place of the OR, the operating bed, so as to assess the air quality near the patient.

Considering that the presence and the number of staff members can influence the air quality, it was decided to allow the presence of 2 staff members in the OR when required by the sampling.

When the staff members were present, they were positioned at last 2 m from the detection equipment, wearing non-woven surgical gowns and surgical masks and with covered feet and hair. The 2 different samplings in the OR (bacterial load and numbers of airborne particles) were performed simultaneously.

Since the pollution variation during the sampling with open doors is related to the air quality in the operating complex, the air quality outside the OR, in the hallway, was also evaluated using the same parameters described above and positioning the sampling equipment at about 2 m from the entrance of each OR.

As mentioned above, it is necessary to wait some time for the experimental conditions until they influence the air pollution. To calculate such time, the following formula was used:

n = [ln (C_0_/C_t_)]/t,

where n = number of air changes, C_0_ = initial concentration, C_t_ = concentration after time t, and t = elapsed time expressed in hour [24].

Considering that in the OR the air exchanges are at least 15 Vol/hr, to achieve a theoretic value of about 99% of the final value, it wass necessary to wait about 30 min until the air changed in the new condition. Therefore, after the implementation of the new experimental condition, it was decided to wait at least 30 min before starting our sampling.

The sampling was conducted in a condition of inactivity of the OR and at least 30 min after routine cleaning protocols. Measurements were performed under the following conditions:

- An empty OR with equipment positioned 30 min before monitoring, after all staff members left, and with closed doors;
- In the presence of staff members wearing the previously described clothing and surgical masks, 30 min after they entered the OR, and with closed doors;
- In the presence of staff members 30 min after the removal of surgical masks and with closed doors;
- 30 min after the doors were opened and without staff members;
- In the hallway outside the OR.

### 2.4. Statistical Analysis

Pollutant concentrations were described using geometric means with range (min to max); the comparison among the different experimental conditions was based on a two-way ANOVA on the log-transformed values, using aerodynamic diameter as between factor and the different OR conditions as within factor. Pairwise comparisons were based on the Student’s t-test for paired samples.

Microbial load was synthetized using median and range and compared among the different experimental conditions using Friedman test followed by the Wilcoxon test for paired sample. 

In all the analyses, multiplicity issue was addressed using the Holm correction [25], and *p*-values < 0.05 denoted statistical significance.

Statistical analysis was performed using the statistical platform R version 4.0.1 [26].

## 3. Results and Discussion

### 3.1. Influence of Monitored Factors on 5 µm and 10 µm Particles

Table 1 reports geometric means with range of pollutants concentration in each examined condition and according to the aerodynamics diameter of airborne particles.

In all measurements, the count of 5 µm particles was lower than the limit imposed by ISO 14644-1:2015 regulation in order to be classified in the ISO 7 class. The number of air changes was higher than the minimum required by the regulation (15 Vol/h) for all but one OR. 

In the ANOVA analysis, a significant two-way interaction between the diameter and OR condition was observed (*p* = 0.009). 

**Figure 1:**
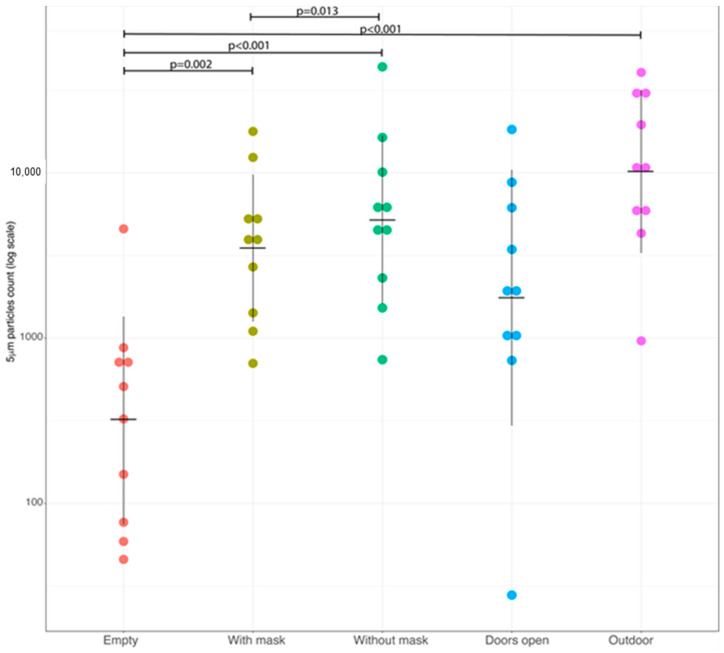
Dot plot showing concentration of airborne particles with diameter ≥5 µm for each of the experimental conditions. Each dot represents a single measurement on log scale. Horizontal lines refer to geometric mean and vertical lines denote ±1 standard deviation of log values. The *p*-values were obtained using pairwise comparison based on a Student’s t-test for paired samples after the application of the ANOVA omnibus test. Multiplicity issue was addressed using Holm correction.

In particular, with respect to airborne particles with an aerodynamic diameter ≥5 µm (Figure 1), a significantly lower concentration of pollutants was observed when comparing the base condition (ORs without operators and with closed doors) with those characterized by the presence of operators either wearing (*p* = 0.002) or not wearing (*p* < 0.001) surgical masks and in the case of measurements made in the corridor outside the OR (*p* < 0.001). The base condition, instead, did not show significant differences with respect to a condition without operators but with open doors. Furthermore, the presence of operators wearing surgical masks was associated with a significantly lower concentration of pollutants with respect to the condition where operators did not wear surgical masks (*p* = 0.013).

Therefore, for the 5 µm particles, their concentrations in the OR significantly changed compared to the empty OR when staff members with or without surgical masks were present in the room. No variation associated with opened or closed doors was detected.

In addition, no significant variation was detected between an OR with closed doors and staff members wearing or not wearing surgical masks in comparison to an empty OR with opened doors. 

Different values, on the other hand, were observed when comparing the use or not of surgical masks. 

**Figure 2:**
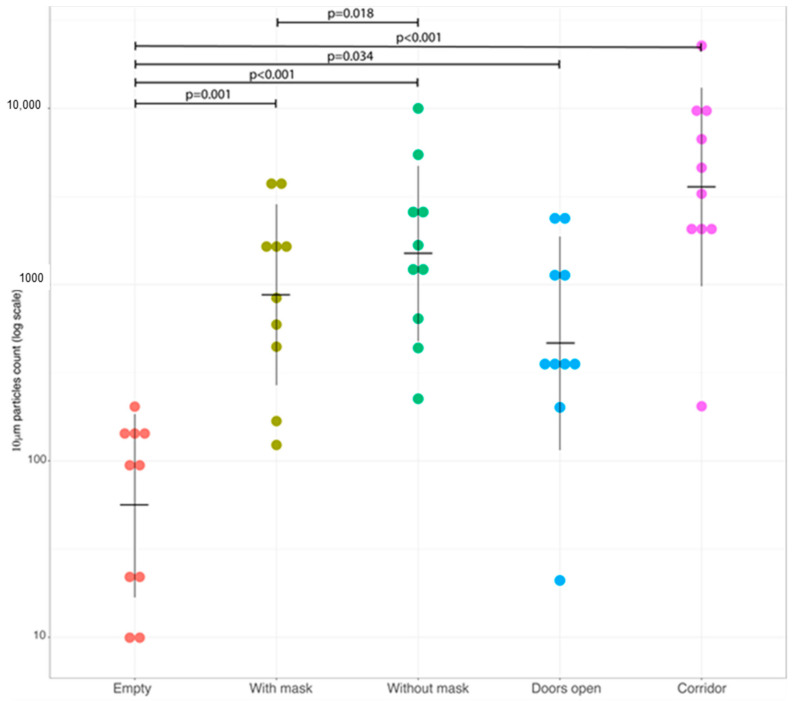
Dot plot showing concentration of airborne particles with diameter ≥10 µm for each of the experimental conditions. Each dot represents a single measurement on log scale. Horizontal lines refer to geometric mean and vertical lines denote ±1 standard deviation of log values. The *p*-values were obtained using pairwise comparison based on the Student’s t-test for paired samples after the application of the ANOVA omnibus test. Multiplicity issue was addressed using Holm correction.

When we considered airborne particles with an aerodynamic diameter ≥10 µm (Figure 2), a similar pattern was observed in comparisons involving the base condition but with an additional significant difference showing a lower concentration also with respect to measurements made in case of open doors (*p* = 0.034). Even with larger particles, wearing a mask significantly reduced the pollutant concentration (*p* = 0.018). No differences were observed comparing conditions with the presence of operators (either wearing or not wearing a surgical mask) and conditions without operators and open doors.

Therefore, for 10 µm particles, their concentration significantly changed for all tested conditions (presence or not of staff members with or without surgical masks and always open doors) when compared to an empty OR. A significant difference was also observed when we compared the use or not of surgical masks.

For these particles, we did not observe a significant variation between the following experimental conditions: (a) an OR with staff members wearing or not wearing surgical masks and a closed door and (b) an empty OR with opened doors.

Several studies monitored the presence of particles in ORs. For example, Della Camera et al. measured PM in operating theatres at the University Hospital of Siena, Italy, in the situation of both closed doors and main doors closed/open twice a minute. The study showed that when the doors were opened, PM was influenced by the ventilation system and the design of the room. The variations in PM were greater in rooms with turbulent flow than in those with laminar flow [27].

Regarding the opening of doors, a study by Weiser et al. observed that opening only one door did not allow for a change in positive pressure in the OR while the opening of two doors allowed contaminated air to enter the room [28].

This study showed that keeping opened doors with an empty OR induced a higher concentration of 10 µm particles. It is relevant to remember that in the ORs, there is an input of fresh air from the central part of the ceiling, right above the surgical bed where our sampling equipment was positioned, while the contaminated air is removed from the room through nozzles positioned on the lower part of the walls.

The authors can hypothesise that this centrifugal flow might be an obstacle for the more contaminated air coming from the hallway to reach the sampling area. This mechanism has a minor effect as the aerodynamic diameter of the suspended particles increases, so it would be less effective on 10 µm particles.

This mechanism could be highly efficient if we consider that the pollutant concentration in the hallways is more than a higher order of magnitude compared to the empty OR for the particles and more than three fold higher for the TVC.

The pollution created by the presence of two staff members, whether or not they were wearing masks, was not significantly different from that due to the opened doors when the OR is empty. Evidently, considering the mechanism previously mentioned, the presence of people in the room produces an amount of pollutants comparable to those entering the OR when the doors are kept opened.

### 3.2. Influence of Monitored Factors on Microbial Load

The median microbial load, according to the OR conditions, is reported in Table 2; The Friedman test showed a significant effect of the OR’s condition on the microbial load (*p* < 0.001). 

**Figure 3:**
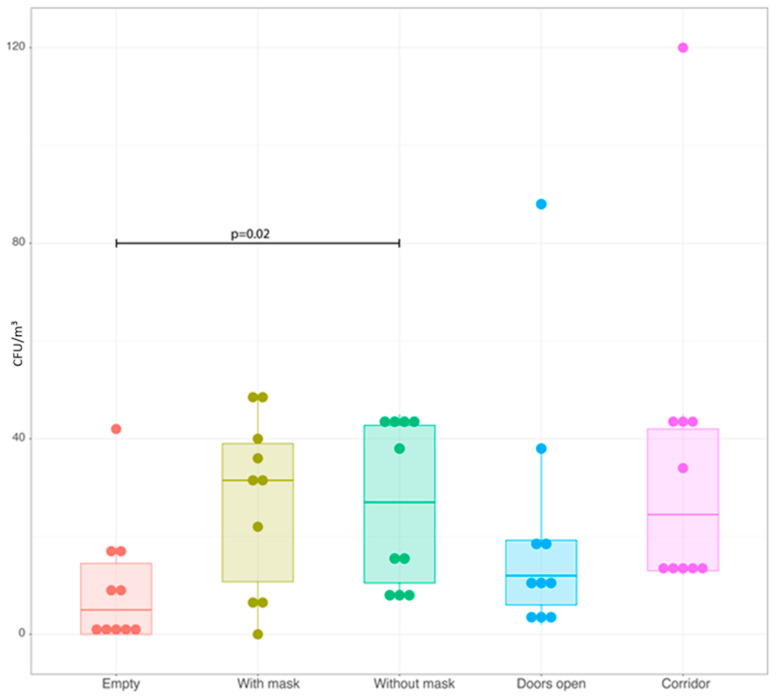
Box-plot showing TVC for each of the experimental conditions. Each dot represents a single measurement. Boxes are defined by first quartile (Q1), Median and third quartile (Q3). Whiskers reach the minimum and the maximum of the distribution except for the presence of outliers, defined as data points below Q1−1.5*(Q3−Q1) or above Q3 + 1.5*(Q3−Q1). The *p*-values were obtained using pairwise comparison based on the Wilcoxon test for paired sample after the application of the Friedman omnibus test. Multiplicity issue was addressed using Holm correction.

After adjusting for multiple comparison, a significant difference (*p* = 0.02) was observed only when comparing the base condition with measurements made when operators did not wear surgical masks. Interestingly, no significant differences were reported between the condition in which people were wearing or not wearing surgical masks (Figure 3).

Therefore, no change in the observed TVC was registered in different experimental conditions, except when staff members who were not wearing surgical masks were present compared to an empty OR. It is important to notice that the use or not of surgical masks showed no significant difference. The same applies for the opening or not of the doors.

The microbial load in the ORs was analysed in several occasions. For example, regarding the use of masks, the study by Zhiqing et al. assessed the bacterial contamination present on surgical masks used or not used by surgeons in the OR. The results showed a higher bacterial contamination on surgical masks worn by surgeons than in those not used, suggesting that a possible source of contamination may be the surgeon rather than the OR environment [29]. Edmiston et al., on the other hand, assessed nasopharyngeal microbial shedding from subjects wearing or not wearing a mask. The study revealed a significant difference in microbial shedding at 90 min of mask wearing between the two groups, but not at 3 h. The study showed, therefore, that the barrier action of the mask reduced over time and suggested changing the mask after 60–90 min intervals [30].

Other studies that analysed air quality in ORs were those of Pasquarella et al., who monitored the air quality in the OR during an operation both when the staff behaved correctly and when they did not comply with the recommendations, revealing greater particle and microbiological air contamination in the latter case [31].

With regard to the presence of people in ORs, a study by Sadrizadeh et al. calculated the influence of the number of people on bacterial carrier particles (BCPs). They assessed that as the number of people increased, BCPs tended to increase [32].

The presence in the OR of staff members wearing or not wearing surgical masks induces a generic increase of pollutant concentrations. The use or not of surgical masks induces an increase in the pollutant concentrations, but not in the TVC.

The authors posit that these results can be explained by the distance of staff members from the sampling (2 m). At such distance, the smallest drops and the aerosol produced by the respiratory tract containing microorganisms may evaporate while drops with a bigger diameter fall to the floor before reaching the sampling point for gravity.

In addition, several studies detected bacterial contamination in air samples [33,34,35,36,37] and water samples [38,39,40] in Italian healthcare facilities.

In our study, we used active air sampling by SAS Super ISO 180.

Air sampling can be carried out in two methods: active and passive. Active air sampling measures the concentration of viable particles while passive air sampling measures the rate at which these particles deposit on surfaces [41]. A study by Pasquarella et al. evaluated bacterial and fungal contamination in an Italian University Hospital using the two types of sampling, showing a significant correlation between the two methods for bacterial contamination, but a higher fungal identification efficiency for active rather than passive sampling [41]. 

## 4. Conclusions

Studying the air pollution in ORs during surgeries is particularly difficult for different reasons, such as the differences in surgical procedures, the variability of the conditions in which such surgeries take place, the number of staff members in the room, the movements executed by staff members, and so on.

Moreover, during a single surgery, generally, such conditions can rapidly change. Since the effects due to a single variation need a specific time to affect the air pollution, it is possible to generate an overlapping of such effects. For this reason the current study was carried out in ORs not in use and using a specific protocol that included the variation of experimental conditions one at time and taking into consideration the necessary time to achieve a stationary condition for the air pollutants before introducing a new modification.

The results of this study showed that there was a large variability in all experimental conditions, and for this reason, it is wise to look at the observed results as a trend.

Hence, we can definitively say that from the analysis of our data, we can deduce a series of results (mechanisms of which are presented in the “results and discussion” section).

A factor particularly effecting the air pollution is the presence of people in the OR despite wearing proper attire. This can actually be considered the most polluting among those factors considered. With only two staff members, the increase of dust and airborne microorganisms is significant. Hence it is possible to postulate that an even higher increase could be induced by the presence of more staff members and patients and with the use of different equipments and materials.

The TVC did not significantly change with the use of surgical masks by staff members situated at a distance of 2 m. We could suggest that the use of masks beyond a determined distance is not necessary in normal conditions of use. This will support the decision in some hospitals for a less prolonged use of surgical mask by anaesthesiologists, but keeping the mandatory use for surgeons and their assistants. Few and contrasting studies have been published on the use of surgical masks to prevent post-surgery infections [42,43,44,45].

Keeping OR doors open compared to an empty OR did not induce a significant increase of TVC and of 5 μm particles, but only of the 10 μm ones.

The effect of the presence of the two staff members, with or without wearing a surgical mask, with the door closed is not significantly different from that produced by the air input in the empty OR when doors are kept open.

All these results suggest that, for the described mechanisms relative to the air flows present in the OR, the pollutants present in the hallways are not able to significantly reach the center of the room where the operating bed is positioned; therefore, during a surgery in the presence of a higher number of staff members and a patient, with the actual ventilation systems, keeping the doors opened would not induce a significant increase in air pollution.

If we suppose a correlation between the number of people in the room and air pollution deriving from them, we could even assume that with a higher number of people present in the room it would be better to keep the door opened.

Therefore, the main cause of air pollution in an OR seems to be the presence of staff members.

In conclusion, our results clarified the role of some environmental and behavioral factors on the air pollution in the OR.

## Figures and Tables

**Table 1:** Geometric means of airborne particles count according to their aerodynamic diameter and the OR’s conditions.

	5 μm ^1^	10 μm ^2^
**Empty (A)**	318 [46 to 4580]	55.7 [9 to 203]
**With mask**	3507 [703 to 17,800]	876 [123 to 4160]
**Without mask**	5207 [740 to 43,800]	1498 [225 to 10,000]
**Doors open**	1755 [28 to 18,300]	464 [21 to 2507]
**Corridor**	10,200 [962 to 40,500]	3582 [204 to 22,700]

^1^ 5 μm: Particles count 5 μm [min to max]; ^2^ 10 μm: Particles count 10 μm [min to max].

**Table 2:** Median with range of microbial load according to the OR’s conditions.

	CFU/m^3 3^
**Empty**	5 [0 to 42]
**With mask**	31.5 [0 to 49]
**Without mask**	27 [7 to 45]
**Doors open**	12 [2 to 88]
**Corridor**	24.5 [12 to 120]

^3^ CFU/m^3^: Value of median of CFU/m^3^ with range [min to max] of TVC.

## Data Availability

The datasets obtained and analyzed in the current study are available from the corresponding author on reasonable request.
